# DNA methylation signature associated with Bohring-Opitz syndrome: a new tool for functional classification of variants in ASXL genes

**DOI:** 10.1038/s41431-022-01083-0

**Published:** 2022-04-01

**Authors:** Zain Awamleh, Eric Chater-Diehl, Sanaa Choufani, Elizabeth Wei, Rebecca R. Kianmahd, Anna Yu, Lauren Chad, Gregory Costain, Wen-Hann Tan, Stephen W. Scherer, Valerie A. Arboleda, Bianca E. Russell, Rosanna Weksberg

**Affiliations:** 1 Genetics and Genome Biology Program, Research Institute, The Hospital for Sick Children, Toronto, ON Canada; 2 Department of Pediatrics, Division of Genetics, David Geffen School of Medicine, University of California, Los Angeles, CA USA; 3 Division of Clinical & Metabolic Genetics, The Hospital for Sick Children, Toronto, ON Canada; 4 Department of Paediatrics, University of Toronto, Toronto, ON Canada; 5 Department of Molecular Genetics, University of Toronto, Ontario, ON Canada; 6 Division of Genetics and Genomics, Boston Children’s Hospital, Boston, MA USA; 7 Institute of Medical Sciences, University of Toronto, Toronto, ON Canada; 8 Department of Human Genetics, David Geffen School of Medicine, University of California, Los Angeles, CA USA; 9 Department of Pathology & Laboratory Medicine, David Geffen School of Medicine, University of California, Los Angeles, CA USA; 10 Department of Computational Medicine, University of California, Los Angeles, CA USA

**Keywords:** DNA methylation, Neurodevelopmental disorders

## Abstract

The additional sex combs-like (ASXL) gene family—encoded by *ASXL1*, *ASXL2*, and *ASXL3*—is crucial for mammalian development. Pathogenic variants in the *ASXL* gene family are associated with three phenotypically distinct neurodevelopmental syndromes. Our previous work has shown that syndromic conditions caused by pathogenic variants in epigenetic regulatory genes show consistent patterns of genome-wide DNA methylation (DNAm) alterations, i.e., DNAm signatures in peripheral blood. Given the role of ASXL1 in chromatin modification, we hypothesized that pathogenic *ASXL1* variants underlying Bohring-Opitz syndrome (BOS) have a unique DNAm signature. We profiled whole-blood DNAm for 17 *ASXL1* variants, and 35 sex- and age-matched typically developing individuals, using Illumina’s Infinium EPIC array. We identified 763 differentially methylated CpG sites in individuals with BOS. Differentially methylated sites overlapped 323 unique genes, including *HOXA5* and *HOXB4*, supporting the functional relevance of DNAm signatures. We used a machine-learning classification model based on the BOS DNAm signature to classify variants of uncertain significance in *ASXL1*, as well as pathogenic *ASXL2* and *ASXL3* variants. The DNAm profile of one individual with the *ASXL2* variant was BOS-like, whereas the DNAm profiles of three individuals with *ASXL3* variants were control-like. We also used Horvath’s epigenetic clock, which showed acceleration in DNAm age in individuals with pathogenic *ASXL1* variants, and the individual with the pathogenic *ASXL2* variant, but not in individuals with *ASXL3* variants. These studies enhance our understanding of the epigenetic dysregulation underpinning *ASXL* gene family-associated syndromes.

## Introduction

The additional sex combs-like (ASXL) gene family includes *ASXL1*, *ASXL2*, and *ASXL3* which encode epigenetic scaffolding proteins [1, 2]. Pathogenic variants in *ASXL* genes cause three distinct chromatinopathies that present clinically as neurodevelopmental disorders (NDDs) [2]. Bohring-Opitz syndrome (BOS; [MIM# 605039]) is caused by autosomal dominant truncating variants in *ASXL1* and was molecularly defined in 2011 [3, 4]. BOS is characterized by poor growth, microcephaly, dysmorphic facial features, typical “BOS posture”, variable but typically severe to profound intellectual disability, feeding difficulties, and seizures [4, 5]. More recently, germline pathogenic variants in *ASXL2* (reported in 2016) and *ASXL3* (reported in 2013) were identified as the causes for Shashi-Pena syndrome (SHAPNS; MIM# 617190]) and Bainbridge-Ropers syndrome (BRS; MIM# 615485), respectively [6, 7].

Somatic truncating variants in *ASXL1* are associated with age-related clonal hematopoiesis as well as various cancers [8]. Individuals with BOS have been reported to have Wilms tumor, suggesting that germline *ASXL1* mutations increase the risk for certain cancers [1, 4]. The clinical interpretation of *ASXL1* variants can be challenging, particularly when relying on population databases such as the genome aggregation database (gnomAD) [9]. Pathogenic variants in *ASXL1* are reported at higher frequencies in the population than expected, partly due to acquired somatic mosaicism during hematopoietic clonal expansion which occurs in aging healthy individuals. Current tools for variant interpretation can introduce further ambiguity, emphasizing the need for robust and orthogonal tools for variant classification.

In 1998, studies in Drosophila demonstrated that the *Asx* gene family regulates transcription of developmentally vital genes such as the *Hox* gene cluster [1, 8]. Later studies showed the *ASXL* gene family has a more extensive role in epigenetic and transcriptional regulation [8]. In humans, ASXL1 is expressed in all tissues and interacts with the BRCA1-associated protein 1 (BAP1) to form the polycomb repressive deubiquitination complex (PR-DUB) [10]. The PR-DUB complex deubiquitinates histone 2A lysine 119 (H2AK119ub), a mark deposited by the Polycomb Repressive Complex (PRC1) [10]. Accumulating evidence also suggests a role for ASXL1 in regulating H3K27 trimethylation through interactions with components of the PRC2 [11]. More recent data show that ASXL1 interacts with all members of the cohesin complex required for chromatid separation and transcriptional regulation [12], further expanding the catalog of ASXL1 transcriptional and epigenetic co-regulators.

Our research group and others have shown that neurodevelopmental disorders caused by pathogenic variants in genes encoding epigenetic regulators can be associated with genome-wide changes in DNA methylation (DNAm), termed “DNAm signatures” [13–19]. To date, DNAm signatures for more than > 50 disorders have been defined. Most of these disorders are caused by variants in genes encoding histone modifying enzymes. These DNAm signatures are likely established via crosstalk between histone modifications and DNA methylation. Although the exact mechanisms underpinning DNAm signatures are not yet fully elucidated [20], a rapidly expanding body of work has emerged demonstrating that DNAm signatures have diagnostic utility in classifying variants of uncertain significance (VUS) [13–19].

For VUS classification, the DNAm profile for a single case is compared to a gene-specific DNAm signature. This single case analysis can be extended to compare DNAm profiles of individuals with sequence variants in functionally overlapping genes or other phenotypically overlapping neurodevelopmental disorders [13–19]. For example, the Weaver syndrome DNAm signature not only positively classifies individuals with pathogenic variants in *EZH2* but also those with pathogenic variants in *EED* and *SUZ12* [17]. These three genes encode components of the polycomb repressive complex 2 (PRC2) and are associated with clinically overlapping syndromes. In contrast to VUS classification, the derivation of a robust DNAm signature requires DNA from a cohort of individuals with a specific clinical diagnosis as well as pathogenic variants in the associated gene [21].

Here, we generated a unique DNAm signature for pathogenic *ASXL1* variants in a cohort of individuals with BOS. We then used this signature to classify *ASXL1* VUS and a small number of available pathogenic variants in *ASXL2* and *ASXL3*. The *ASXL2* variant had an overlapping DNAm profile with *ASXL1* variants, defining significant congruence of epigenetic dysregulation for pathogenic variants in *ASXL1* and *ASXL2* but not *ASXL3* variants. We also identified increased epigenetic age acceleration in individuals with pathogenic *ASXL1* variants.

## Methods

### Research participants

Individuals were recruited through a patient registry at The University of California, Los Angeles USA in collaboration with the ARRE (ASXL Rare Research Endowment). Individuals with missense *ASXL1* variants were identified through MSSNG [22], the largest whole genome sequencing [WGS] project for Autism Spectrum Disorder (ASD); SFARI [23] (Simons Foundation Autism Research Initiative) and the Simons Simplex Collection (SSC) using the Genotypes and Phenotypes in Families (GPF) tool (https://gpf.sfari.org/). We identified and recruited 17 individuals carrying *ASXL1* variants, 1 individual carrying an *ASXL2* variant, and 3 individuals carrying *ASXL3* variants. Participant’s demographic, clinical phenotype, and variant information are in Table S1. We split individuals with classic features of BOS and pathogenic variants in *ASXL1* (*n* = 14) into DNAm signature discovery *n* = 8 and validation *n* = 6 cohorts. In the *ASXL1* cohort, three (*n* = 3) individuals carried missense VUS that were included for classification. We also included individuals with truncating variants in *ASXL2* (*n* = 1) and *ASXL3* (*n* = 3) for classification. Banked DNA samples from age- and sex-matched typically developing individuals (*n* = 135) were included as control subjects. These individuals were recruited from the Hospital for Sick Children and the Province of Ontario Neurodevelopmental Disorders (POND) Network and were deemed typically developing (Dr. Gregory Hanna). “Typically developing” was defined as healthy and developmentally normal by using formal cognitive/behavioral assessments (POND) or via physician/parental screening questionnaires (SickKids).

### DNA methylation profiling and data processing

Genomic DNA was extracted from peripheral blood and bisulfite converted using the EpiTect Bisulfite Kit (EpiTect PLUS Bisulfite Kit, QIAGEN). Sodium bisulfite converted DNA was then hybridized to the Illumina Infinium Human Methylation EPIC BeadChip to interrogate more than 850,000 CpG sites in the human genome at The Center for Applied Genomics (TCAG), Hospital for Sick Children Research Institute. Samples were run in a single batch to reduce batch effects. On each microarray chip, cases and controls were randomly assigned a chip position. The *minfi* Bioconductor package in R was used to preprocess data including quality control, Illumina normalization and background subtraction, followed by extraction of beta (β) values [24]. Standard quality control metrics in *minfi* were used, including median intensity QC plots, density plots, and control probe plots; three *ASXL1* samples (EX10, EX11, EX13) had lower median channel intensity values than recommended by *minfi* standards, and were used for signature validation but not discovery. Probes with detection flaws (*n* = 1061), probes near SNPs with minor allele frequencies above 1% (*n* = 29,958), cross-reactive probes (*n* = 41,975) [25], probes with raw beta of 0 or 1 in >0.25% of samples (n = 247), non-CpG probes (*n* = 2,925), and X and Y chromosome probes (*n* = 19,627) were removed, resulting in a total of *n* = 774,051 probes remained for differential methylation analysis.

### DNA methylation age estimation

DNA methylation age (epigenetic age) was estimated using the calculator available for Illumina EPIC assays (http://dnamage.genetics.ucla.edu/) [26]. Estimates of age acceleration were calculated by subtracting the chronological age from the estimated DNAm age. We estimated DNAm age and calculated age acceleration in typically developing controls (*n* = 35), and individuals with variants in *ASXL1* (*n* = 17), *ASXL2* (*n* = 1) and *ASXL3* (*n* = 3). We used a paired Wilcoxon’s test to assess mean differences between chronological and estimated DNAm age in each group. Then to assess whether mean estimates of age acceleration were significantly different between controls and individuals carrying variants in *ASXL* genes we used a Mann-Whitney U-Test.

### DNA methylation signature derivation

To assess DNAm patterns, we identified differentially methylated sites in whole blood-derived DNA from *n* = 8 individuals carrying LOF variants in *ASXL1* and a clinical diagnosis of BOS compared to 26 sex- and age-matched typically developing controls (Table S1 and S2). Sample numbers were not sufficient to generate robust signatures for *ASXL2* or *ASXL3* variants, we instead classified those samples using the generated BOS DNAm signature. For all samples, we applied the blood cell-type proportion estimation tool in *minfi* based on Illumina EPIC array data from FACS-sorted blood cells [26]. We identified differentially methylated CpG sites using *Limma* [27] regression modeling with age, sex, 5/6 cell type proportions (i.e., excluding neutrophils), and DNAm age residual (from Horvath DNAm age calculator) as covariates. The thresholds for differentially methylated CpG sites were Benjamini-Hochberg adjusted *p* value < 0.05 and a|Δβ| > 0.10. Δβ represents the difference in average DNAm (β) between groups. Principal component analysis (PCA) and hierarchical clustering were generated using Qlucore Omics Explorer (QOE, www.qlucore.com).

### Machine learning classification models

We developed a machine learning model using the BOS DNAm signature. Using the R package ‘caret’, CpG sites with correlations equal to or greater than 90% to other signature CpGs were removed as previously described [14]. This led to a set of *n* = 546 non-redundant CpG sites. A support vector machine (SVM) model, trained on the non-redundant CpG sites, was set to “probability” mode to generate SVM scores ranging between 0 and 1 (0%–100%), classifying variants as “high” (score > 0.5) or “low” (score < 0.5). To test model specificity, EPIC array data from additional typically developing controls (*n* = 101) were scored. To test model sensitivity, we classified *n* = 6 validation samples, from individuals with a confirmed LOF variants in *ASXL1* and a clinical BOS diagnosis. We also classified individuals with variants in *ASXL2* (*n* = 1) and *ASXL3* (*n* = 3) to further assess specificity of the model. Lastly, we classified individuals with Kabuki (*n* = 11), Sotos (*n* = 19) and Weaver (*n* = 30) syndromes, carrying pathogenic variants in *KMT2D*, *NSD1*, and *EZH2*, respectively.

### Gene ontology analysis

The list of CpG positions comprising the DNAm signature was submitted to GREAT (Genomic Regions Enrichment of Annotations Tool) for gene ontology (GO) enrichment analysis [28]. Enrichment of each GO term within the gene list was calculated using a foreground/background hypergeometric test over genomic regions, using the set of CpG sites after *minfi* probe quality control (*n* = 774,051) as a background set. Overlapping genes were mapped using default GREAT settings with the following exceptions: the cut-off to annotate a CpG as overlapping with a gene (“distal gene mapping” setting) was set to 10 kb, and only enriched terms with three or more gene hits and FDR < 0.05 were reported. We predicted proteins bound to genomic loci using the ChIP-seq Atlas Enrichment Tool (https://chip-atlas.org/) [29].

## Results

### Molecular Genetics

**Fig. 1:**
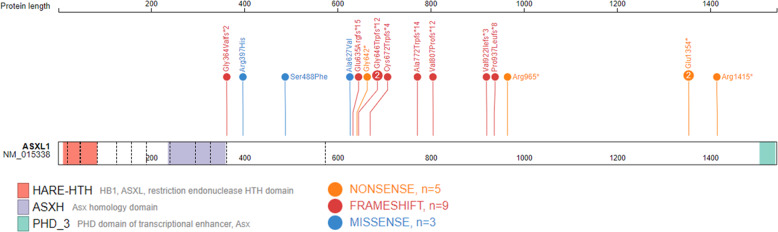
Genomic Location of *ASXL1* variants. Schematic representation of the ASXL1 protein (GenBank: ASXL1; NM_015338.6; GRCh37), its functional domains, and variants used in this study. Exon structure, based on GenBank: NM_015338.6, is provided by dashed lines. Red, HB1, ASXL, restriction endonuclease HTH domain (HARE-HTH, 11–83); purple, Asx homology domain (ASXH, 236–359); green, C-terminal plant homeodomain (PHD, 1506–1539). The N-terminal HARE-HTH domain is DNA binding and with the ASXH domain are required for interaction with BAP1 and NCOA1. The c-terminal PHD is required for interaction with nuclear receptors. The map was generated using ProteinPaint [31].

In this study, we reported 14 individuals with *ASXL1* (NM015338.5) variants that are predicted to adversely impact protein function and are classified as pathogenic using the ACMG variant classification guidelines [30] (Table 1 and S1). Variants reported are frameshift or nonsense located within the last two exons (11 and 12), the observed mutational hotspot in *ASXL1*. Variants in these individuals are associated with the phenotypes of Bohring-Opitz syndrome. The remaining variants (*n* = 3) in *ASXL1* are missense variants of uncertain significance also located within the last two exons. Figure 1 provides a schematic ASXL1 structure and variant location generated using ProteinPaint [31].

**Table 1:** Demographic and variants information for individuals with truncating *ASXL1* variants and a clinical diagnosis of Bohring-Opitz syndrome used for DNAm signature discovery and validation.

Sample_ID	Sex	Age at blood collection (years)	cDNA change	Protein Change	Group
EX1	M	8	c.1924G> T	p. Gly642*	Discovery
EX2	F	15	c.2013_2014del	p. Cys672Trpfs*4	Discovery
EX3	F	10	c.2893C > T	p. Arg965*	Discovery
EX4	M	31	c.1091delG	p. Gly364Valfs*2	Discovery
EX5	M	15	c.2416_2417dupAC	p. Val807Profs*12	Discovery
EX6	M	4	c.4243C > T	p. Arg1415*	Discovery
EX7	F	4	c.4060G > T	p. Glu1354*	Discovery
EX8	F	29	c.2313_2318delinsTTGG	p. Ala772Trpfs*14	Discovery
EX9	M	7	c.2759_2762dupCATC	p. Val922Ilefs*3	Validation
EX10	M	12	c.4060G > T	p. Glu1354*	Validation
EX11	F	9	c.2810delC	p. P937Lfs*8	Validation
EX12	M	2	c.1934dupG	p. G646Wfs*12	Validation
EX13	M	24	c.1910_1922del	p. Glu635Argfs*15	Validation
EX14	F	2 days	c.1934dupG	p. G646Wfs*12	Validation

### Epigenetic age in individuals with *ASXL* variants

**Fig. 2:**
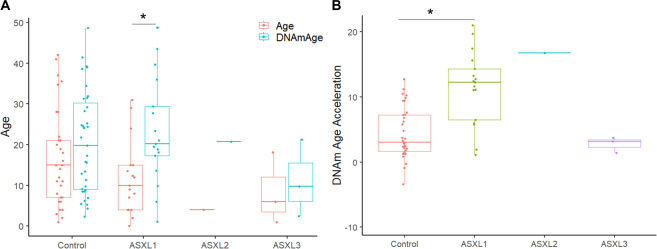
Individuals with *ASXL1* variants exhibit altered epigenetic aging. **A** Box plot comparing “DNA methylation age” (blue) derived from the Illumina 850 K data and reported chronological age (red), on the y-axis. Groups are indicated on the x-axis and include typically developing controls (*n* = 35), individuals with *ASXL1* variants (*n* = 17), individual with *ASXL2* variant (*n* = 1), and individuals with *ASXL3* variants (n = 3). Each individual observation is plotted as a circle. To assess whether the mean difference between DNAm age and chronological age is statistically significant within each group we used a paired Wilcoxon test (*p-value < 0.05). (**B**) Box plot of epigenetic age acceleration (y-axis) obtained by subtracting the chronological age from the estimated DNAm age for each individual. To assess whether mean epigenetic age acceleration estimates are significantly different between controls and individuals carrying variants in *ASXL* genes we used a Mann-Whitney U-Test, except for *ASXL2* with a *n* = 1.

Prior to generating a DNAm signature for *ASXL1*, we used the Horvath [26] DNAm age clock, to estimate epigenetic age for 35 typically developing controls and 21 individuals carrying variants in *ASXL* genes. Mean estimated DNAm age compared to mean chronological age was increased across all groups, however most significantly in individuals with *ASXL1* variants (*p*-value = 3.8e−6) (Fig. 2A). We calculated DNAm (epigenetic) age acceleration by subtracting the chronological age from the estimated DNA methylation age. Mean epigenetic age acceleration was significantly increased in individuals carrying *ASXL1* variants compared to typically developing controls (p-value = 1.1e−7; Mann-Whitney U-Test) (Fig. 2B). Two individuals carrying *ASXL1* variants displayed the lowest age acceleration that also fell within the control range, and they were: subject EX17 (+1.11 years) with a missense variant p. (Ala627Val), and subject EX9 (+1.95 years) a 2-day old female with a frameshift variant p. (Gly696Argfs*11). The remaining two individuals with missense *ASXL1* variants, EX15 and EX16, displayed increased epigenetic age acceleration (+6.5 and +5.9 years), but lower than the average age acceleration observed in individuals with pathogenic *ASXL1* variants and BOS (+14.4 years). The individual with the *ASXL2* variant displayed increased DNAm age and epigenetic age acceleration (+16.7 years) outside of the control range (Fig. 2). Lastly, the three individuals with *ASXL3* variants showed no epigenetic age acceleration and were all within range of typically developing controls.

### Bohring-Opitz Syndrome (BOS) DNAm signature generation

To generate a BOS-specific DNAm signature, we profiled genome wide DNAm in blood from individuals with a confirmed BOS diagnosis due to pathogenic *ASXL1* variants (*n* = 14; Table 1). The BOS discovery cohort (*n* = 8) included 4 females and 4 males with mean age at sample collection of 14.5 ± 10.5 years (range 4–31 years). The 26 sex- and age- matched control subjects included 13 females and 13 males and mean age at sample collection of 17.7 ± 9.4 years (range 4–35 years) (Table S2).

**Fig. 3:**
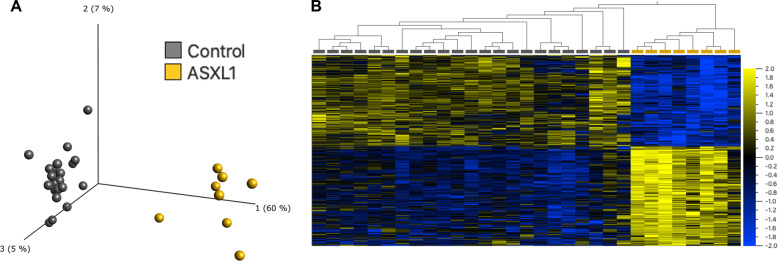
Loss-of-function variants in *ASXL1* are associated with a distinct DNAm signature. **A** Principal component analysis (PCA) and (**B**) heatmap showing clustering of the BOS discovery cohort (*n* = 8; yellow) and control discovery cohort (*n* = 26; grey) using DNAm values at the 763 CpG sites identified in the BOS specific DNAm signature. The heatmap color gradient indicates the normalized DNAm value ranging from −2.0 (blue) to 2.0 (yellow). Euclidean distance metric is used in the heatmap clustering dendrograms.

We identified 763 differentially methylated CpG sites that meet thresholds of FDR < 0.05 and|Δβ| > 0.10 (10% DNAm difference; Table S3), using linear regression modeling. We accounted for DNAm age as a covariate, considering the observed epigenetic age acceleration in individuals with *ASXL1* variants. We visualized DNAm data at signature sites using principal component analysis (PCA) and hierarchal clustering (Fig. 3). DNAm at 763 signature sites clearly distinguished individuals with BOS from typically developing controls; 52% of the signature CpG sites were hypermethylated and 48% were hypomethylated. Approximately 55% of CpG signature sites overlapped islands or shores (within 2 kb of islands), this was significantly higher than the percentage of total probes on the array representing islands and shores (37%) (*p*-value = 9.68E−4; hypergeometric test).

### Ontology of BOS DNAm signature sites

We assessed the ontology of genes overlapping CpG sites in the BOS DNAm signature using GREAT [28]. Using gene ontology analyses we can describe the role of gene targets in three biological domains: molecular-level activity of gene products (molecular functions), larger processes accomplished by multiple molecular functions (biological processes), and phenotypic abnormalities of human disorders that gene targets are predicted to contribute to (human phenotypes). There were 323 unique genes that overlapped 482 of the 763 signature CpG sites, with 86 genes overlapping 2 or more CpG sites. We identified significant enrichment (FDR < 0.05, gene hits ≥ 3) for 46 biological processes, 18 molecular functions, and 105 human phenotypes (Table S4–S6). The top 30 biological processes, ranked based on the number of gene hits, were related to embryonic bone, tissue, and organ development. Driving these biological processes were several *HOX* genes including *HOXA5*, *HOXA11*, and *HOXB4*. The *HOXA5* gene was most notable, overlapping 13 CpG sites in the BOS DNAm signature, all of which were hypermethylated and mapped to a 3 kb window in CpG islands. In this study, we compared DNAm levels at eight of the 13 *HOXA5* CpGs (within ~500 base pairs) in the BOS signature to DNAm values at those same sites in individuals with Kabuki, Weaver, and Sotos syndromes (Figure S1).

Most molecular functions were related to RNA polymerase II transcription factor activity and sequence-specific DNA-binding. The top 20 human phenotypes were related to bone abnormalities of the limbs and limb joints and included gene hits encoding collagens *COL11A1* and *COL6A1*, homeobox proteins *HOXA11*, *HOXA13* and *MEIS1*, and transcription factors *RUNX2* and *TWIST1*. Lastly, we predicted proteins that bind the 763 BOS signature loci and the overlapping histone marks in blood using the ChIP-seq Atlas [29] enrichment tool. Top enriched peaks belonged to transcription factors: TET2, CTCF, the cohesin complex (RAD21, SMC3, SMC1A, and STAG1), and EZH2 from the PRC2 complex, and the top enriched histone mark was H3K27me3 (Table S7).

### Independent validation of BOS DNAm signature

**Fig. 4:**
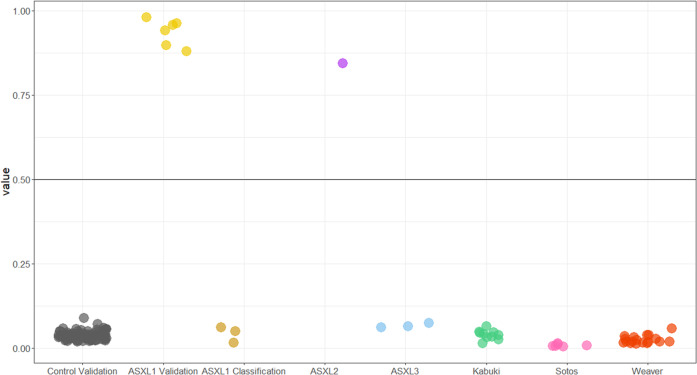
Classification of samples using machine learning models based on the BOS DNAm signature. Sample groups were scored using the BOS support vector machine (SVM) model. The x-axis groups each cohort, and the y-axis shows the probability score. BOS validation subject (*n* = 6) had high probability scores demonstrating 100% sensitivity of the model. Whereas validation control subjects (*n* = 101) all had low scores demonstrating 100% specificity of the model. *ASXL1* missense variants (*n* = 3) and *ASXL3* truncating variants (*n* = 3) scored low similar to controls, whereas the truncating *ASXL2* variant (*n* = 1) scored high similar to the BOS validation group. Lastly, individuals with Sotos, Weaver, and Kabuki syndromes, caused by pathogenic variants in the chromatin-modifying genes *NSD1*, *EZH2*, and *KMT2D* respectively, all scored low further demonstrating 100% specificity of the model. Horizontal line represents threshold for classifying samples as case-like (above line) or control-like (below line).

Using the BOS DNAm signature, we used a machine learning classification model to robustly categorize variants as BOS-like or control-like based on DNAm levels at signature sites. We trained a support vector machine (SVM) model on data from the discovery cohort used to generate the signature which included n = 8 individuals with BOS and typically developing controls (n = 26) (Table 1, S2). The model generated a probability of pathogenicity score from 0 to 1 for each sample, with 0.5 being the decision boundary for classification (Table S8). We classified a validation cohort of six unrelated individuals with a BOS diagnosis and truncating *ASXL1* variants (Table 1). The SVM model generated high pathogenicity scores (87-98%) for the validation cohort demonstrating high sensitivity of the signature (Fig. 4). To test the specificity of the BOS DNAm signature, we included DNAm data for an additional 100 typically developing controls (40% females, and ages 1 to 42 years), all of which had low SVM scores (2–8%) demonstrating high specificity of the signature (Fig. 4).

### Classification of variants in other *ASXL* genes causing neurodevelopmental disorders

The *ASXL* gene family also includes *ASXL2* and *ASXL3*, and variants in these genes are implicated in Shashi-Pena and Bainbridge-Ropers syndromes, respectively [6, 7]. We used the SVM model to classify a truncating germline *ASXL2* variant and three truncating germline *ASXL3* variants (Table S1). The individual with the *ASXL2* variant had a pathogenicity score of 84% on the SVM model and classified with the validation BOS cohort. In contrast, all individuals with truncating *ASXL3* variants, had SVM scores of 6–7% within the range of controls (Table S8 and Fig. 4). We also visualized differences in DNAm for all individuals with *ASXL* variants based on the BOS DNAm signature sites using PCA and hierarchal clustering, and clustering patterns are in line with the SVM model classification (Figure S2).

To further validate the specificity of the BOS DNAm signature generated, we classified three cohorts of individuals with Sotos, Weaver, and Kabuki syndromes, caused by variants in genes encoding the epigenetic regulators, *NSD1*, *EZH2*, and *KMT2D*, respectively [13, 14, 17]. All three cohorts had pathogenicity scores within the control range (1–7%) (Fig. 4).

### *ASXL1* VUS Classification

Having illustrated the efficacy of the BOS DNAm signature in robustly classifying individuals with pathogenic *ASXL1* variants, we next classified three individuals with *de novo* VUS in *ASXL1*. We also assessed predicted pathogenicity of these VUS using Alamut variant annotation software, which applies multiple prediction algorithms for comparison (Table 2). The SVM model for BOS DNAm signature sites generated low pathogenicity scores for all three missense variants (1-6%). For individual EX17 *ASXL1* p. (Ala627Val), the DNAm data are in line with a non-BOS diagnosis. This individual is diagnosed with pervasive developmental disorders (PDD), attention deficit disorder (ADD), and Raynaud disease. This individual exhibits intellectual and speech delay but does not present with any of the hallmark features of BOS such as facial dysmorphism, growth restriction at birth, microcephaly, or recurrent infections. Clinical information available for individuals EX15 *ASXL1* p. (Ser488Phe) and EX16 *ASXL1* p. (Arg397His) was limited. Aside from a diagnosis of autism spectrum disorders (ASD), no other phenotypic information was available.

**Table 2:** Predicted pathogenicity of missense *ASXL1* variants generated using Alamut.

Sample	Protein Change (NM015338.5)	Predicted pathogenicity					
		SIFT (score)	PolyPhen-2	MutationTaster	CADD	SVM Score	DNAm signature classification
EX0789	p. Ala627Val	Tolerated (0.14)	Benign	Polymorphism	24.5	0.015	Negative
EX0731	p. Ser488Phe	Deleterious (0.02)	Probably Damaging	Disease Causing	24.3	0.06	Negative
EX0732	p. Arg397His	Tolerated (0.12)	Benign	Disease Causing	25	0.04	Negative

## Discussion

In this study we profiled genome-wide DNA methylation in peripheral blood from 21 individuals with variants in *ASXL* genes. We used the DNAm data to (i) assess epigenetic age acceleration, (ii) generate a BOS-specific DNAm signature, and (iii) to classify *ASXL1* VUS, and truncating *ASXL2* and *ASXL3* variants based on the unique BOS DNAm signature.

We report epigenetic age acceleration in individuals with BOS, but in not individuals with Bainbridge-Ropers syndrome; however, the latter could be due to a limited sample number (*n* = 3). Epigenetic age acceleration has previously been reported in Sotos syndrome and Tatton-Brown-Rahman syndrome (TBRS), which are caused by loss of function variants in two genes encoding epigenetic regulators, *NSD1* and *DNMT3A*, respectively [32, 33]. *NSD1* encodes a histone H3 lysine 36 (H3K36) methyltransferase responsible for regulating levels of trimethylation (H3K36me3), and *DNMT3A* encodes a *de novo* DNA methyltransferase [13, 33]. DNAm signatures for Sotos and TBRS indicate a primarily hypomethylated DNAm profile compared to typically developing controls with only a small proportion of hypermethylated sites [13, 33]. This aligns with global DNAm patterns observed during physiological aging, where the majority of the DNA is hypomethylated and preferentially hypermethylated at bivalent promoters [34]. This DNAm pattern is not consistent with the BOS DNAm signature we report, which has an approximately even number of hyper- and hypo- methylated CpG sites. While estimating epigenetic age can be an interesting biomarker, we don’t yet fully understand mechanisms driving DNAm changes and how they relate to physiological aging. However, estimating DNAm age in developmental disorders similar to BOS, will contribute to our understanding of DNAm changes related to aging.

Neurodevelopmental syndromes associated with *ASXL* genes are rare and have only recently been described. While the effects of *ASXL* genes variants have been studied in the context of somatic changes and cancer, the effects of germline *ASXL* variants in the context of the neurodevelopmental syndromes have not been well investigated. There are no iPSC models currently available for *ASXL1* variants in BOS, and germline-modified mouse models have high rates of embryonic and postnatal mortality [35]. Most studies investigating effects of somatic *ASXL1* variants are conducted in leukemic cell lines since these variants have been associated with acute myeloid leukemia (AML). Studies targeting *ASXL1* gene expression in vitro have reported dysregulation of the posterior *HOXA* cluster (*HOXA5*, *HOXA7*, *HOXA9)* [11, 36, 37]. Several CpG sites in the BOS DNAm signature overlapped *HOX* genes, most notably 13 hypermethylated CpG sites in *HOXA5*, a homeobox sequence-specific transcription factor with finely tuned expression during development [38]. *HOXA5* is a tumor suppressor gene dysregulated in expression and methylation in several types of cancer [39]. Our DNAm studies in Wilms tumor tissues identified hypermethylation of *HOXA5* CpG sites [40, 41]. This finding is of interest since BOS individuals appear to have an increased risk for Wilms tumor [4]. In our previously published disorder-specific DNAm signatures we identified aberrant *HOXA5* methylation in individuals with Weaver and Kabuki syndromes [14, 17]. In this study, we compared DNAm levels at *HOXA5* CpGs in individuals with BOS, Kabuki, Weaver, and Sotos syndromes (Figure S1). Both individuals with BOS and Kabuki syndrome showed hypermethylation, whereas individuals with Weaver syndrome showed hypomethylation at these sites. Identifying recurrently affected *HOX* gene CpG sites across multiple NDDs defines critical regions that should be followed up by independent experimental approaches.

While research on the *ASXL* gene family has advanced rapidly, the exact mechanisms by which ASXL1 impacts DNA methylation of target genes have not been fully elucidated. In regards to the regulation of the posterior HOXA cluster by ASXL1, there is one proposed mechanism from a study in *ASXL1*^-/-^ myeloid leukemia cells, which showed global depletion of H3K27me3 and EZH2 at the posterior HOXA cluster [11]. The same study reported direct physical interactions between ASXL1 and EZH2, a PRC2 protein responsible for depositing the H3K27me3 mark [11], and a protein that can interact with DNA methyltransferases thus impacting DNA methylation [42]. More recent ChIP-seq analyses of all epigenetic marks identified the co-occurrence of H3K27me3 at 74% of genomic regions with H2AK119ub marks [37]. Using the ChIP-seq Atlas enrichment tool [29], we predicted proteins bound to the 763 genomic loci in the signature and the overlapping histone marks. Interestingly the top enriched histone mark was H3K27me3 and EZH2 was one of the top enriched proteins bound (Table S7). Another potential mechanism by which ASXL1 can impact DNA methylation is through protein interactions with methyl-CpG binding domain proteins 5 and 6 (MBD5 and MBD6) [43]. MBD proteins coordinate crosstalk between DNA methylation, histone modifications, and chromatin organization [44]. These findings indicate that the actions of ASXL1 at a single genomic locus may result in a spectrum of epigenetic and transcriptional responses.

Previous DNAm signature studies in NDDs have contributed to our understanding of functional protein complexes and families. High or intermediate SVM classification of variants in one gene using the signature of another can occur when their protein products interact [1, 8, 21]. In addition to our findings in Weaver syndrome where variants in genes encoding components of PRC2 resulted in an overlapping DNAm profiles, individuals with variants in genes encoding components of the chromatin remodeling BAF complex, had overlapping DNAm profiles. Studies have shown that DNAm profiles at subsets of CpGs overlap between individuals with BAFopathies carrying variants in *SMARCA2* that cause NCBRS, and in *ARID1B*, *SMARCB1*, and *SMARCA4* that lead to Coffin-Siris syndromes type 1, 3, and 4, respectively. With respect to the *ASXL* gene family, phylogenetic analyses of coding sequences showed that ASXL1 and ASXL2 are more closely related than to ASXL3, and while ASXL1 and ASXL2 are expressed in most tissues, ASXL3 is strictly expressed in the brain. Studies have also shown co-immunoprecipitation of ASXL2 protein with BAP1, and that ASXL1 and ASXL2 can form mutually exclusive PR-DUB complexes with BAP1 [45, 46]. In this study, the DNAm profile of the individual with the truncating *ASXL2* variant and a SHAPNS diagnosis had an overlapping DNAm profile to that of individuals with BOS. The individual with SHAPNS had a high pathogenicity SVM score (84%) based on the BOS DNAm signature and displayed epigenetic age acceleration, whereas the three individuals with truncating *ASXL3* variants had low SVM scores (6–7%) and displayed no epigenetic age acceleration. While the overlapping DNAm signal is notable, we acknowledge that we have only one observation and the best practices for DNAm application include using only the gene-specific signature developed to classify VUS. However, *ASXL2* germline variants are extremely rare, in part due to the recent identification of the SHAPNS clinical phenotypes, with only seven individuals reported in the literature [2, 7]. As more cases of *ASXL2* are classified we expect to better define the congruence between the DNAm signatures for pathogenic variants in *ASXL1* and *ASXL2*.

We used the BOS-specific DNAm signature to classify three individuals with *ASXL1* VUS, all of which had pathogenicity SVM scores similar to those in controls. Clinical phenotype of individual EX17 p. (Ala627Val) is inconsistent with a BOS diagnosis, this is in line with the DNAm-negative classification for BOS (Table S1). To further test the specificity of the BOS DNAm signature we classified individuals with three syndromes: Sotos, Weaver, and Kabuki. Similar to *ASXL1* in BOS, these syndromes are caused by variants in chromatin-modifying genes (*NSD1*, *EZH2*, *KMT2D*). Despite commonly dysregulated CpG sites across these syndromes, the BOS DNAm signature differentiated all individuals with these NDDs from those with BOS. This further demonstrates the specificity of the BOS DNAm signature.

In conclusion, we report an increased epigenetic age acceleration in individuals with BOS and a highly sensitive and specific BOS DNAm signature. These findings demonstrate the emerging trend in DNAm signature research, namely their utility beyond classifying VUS, in understanding and characterizing functional relationships between disorders. Future studies should include cell-type-specific multi-omics approaches which will be required to further elucidate molecular and epigenetic changes associated with *ASXL1* variants causing BOS. Such studies will help determine whether there is a direct or indirect functional consequence of ASXL variants on DNAm and will also be useful in defining potential treatment targets for ASXL associated disorders.

## Supplementary information

Supplementary Tables S1–S8

S1

S2

Figure S1 and S2 legends

## Data Availability

The datasets generated during the current study are not publicly available due to institutional ethical restrictions but are available from the corresponding author on reasonable request.
