# Supplementary figures and images for: DNA methylation signature associated with Bohring-Opitz syndrome: a new tool for functional classification of variants in ASXL genes
Source: Eur J Hum Genet. 2022 Apr 1;30(6):695–702. doi: 10.1038/s41431-022-01083-0 (PMC9177544; doi:10.1038/s41431-022-01083-0)

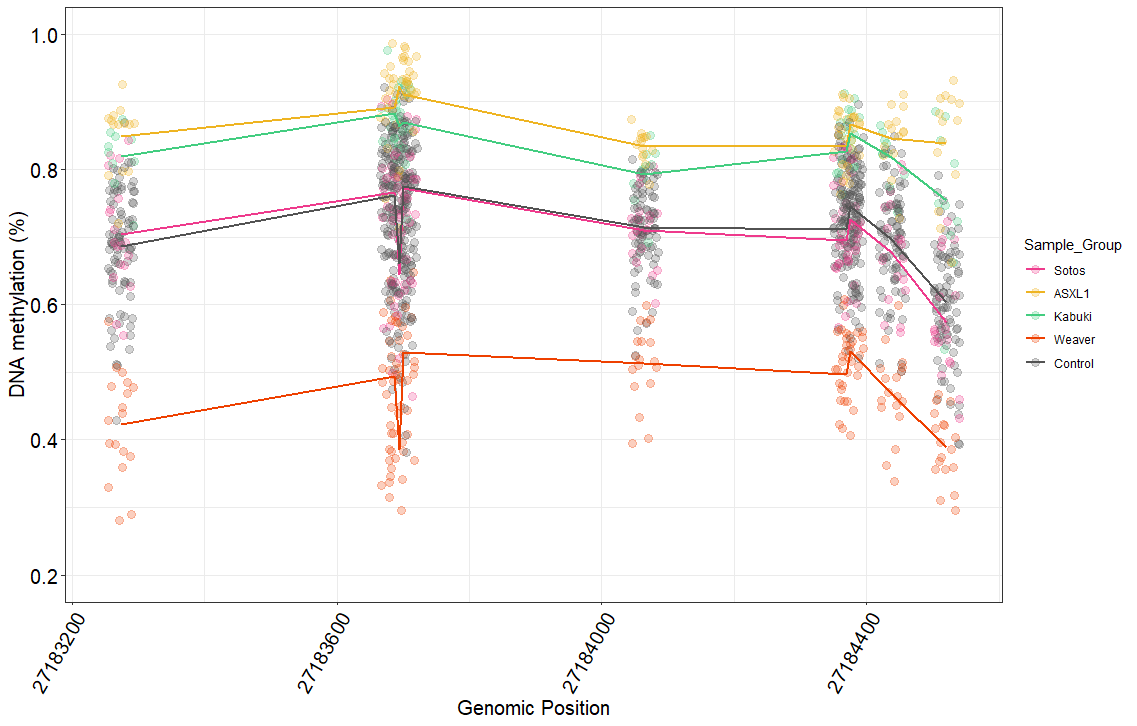

Supplement: Supplementary file 2 — S1 [file 41431_2022_1083_MOESM2_ESM.tif]

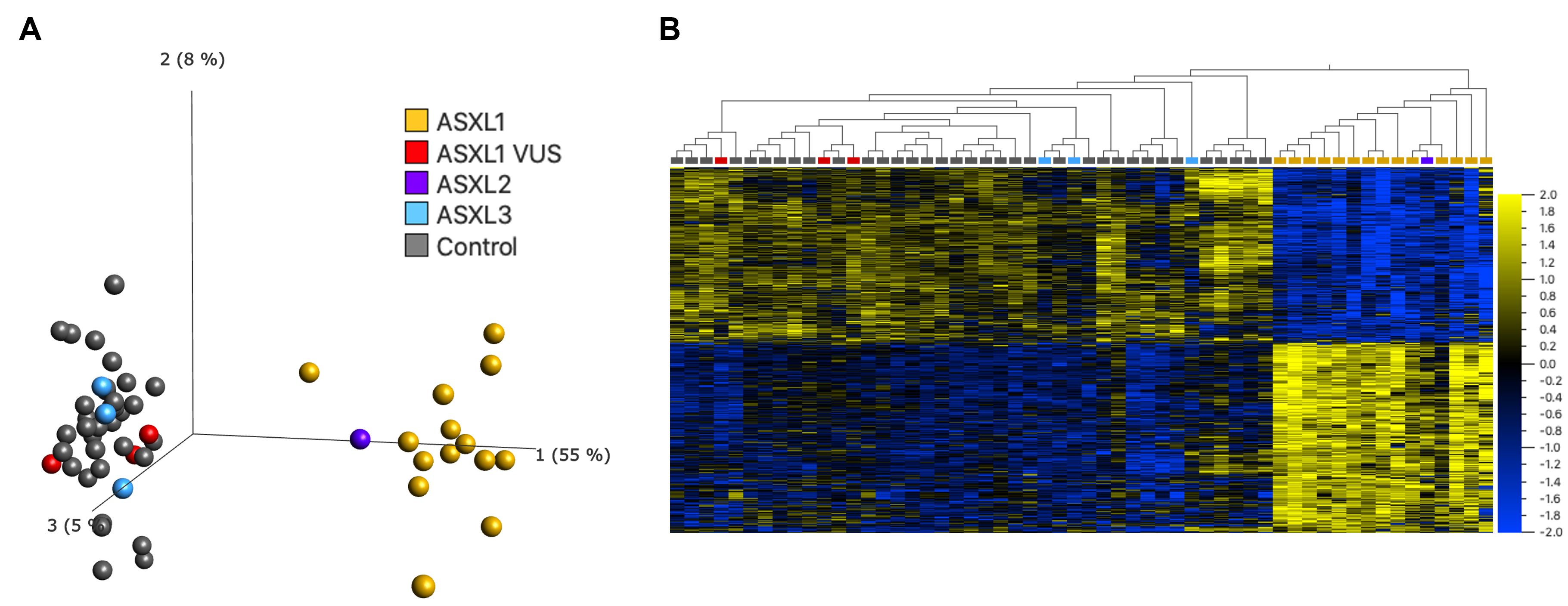

Supplement: Supplementary file 3 — S2 [file 41431_2022_1083_MOESM3_ESM.png]
