# Supplementary material for: DNA methylation signature associated with Bohring-Opitz syndrome: a new tool for functional classification of variants in ASXL genes
Source: Eur J Hum Genet. 2022 Apr 1;30(6):695–702. doi: 10.1038/s41431-022-01083-0 (PMC9177544; doi:10.1038/s41431-022-01083-0)
Supplement: Supplementary file 4 — Figure S1 and S2 legends [file 41431_2022_1083_MOESM4_ESM.docx]

Figure S1. HOXA5 overlapping differentially methylated sites in BOS DNAm signature. Scatter plot of Beta values at 8 of the 13 CpG sites found in the BOS DNAm signature. Methylation values at the CpG sites are shown for typically developing controls (n=35), individuals with BOS (n=14), Sotos (n=6), Weaver (n=18), and Kabuki syndromes (n=6). The lines connect the average beta values for each corresponding group at each CpG site. Across all CpG sites individuals with BOS and Kabuki were hypermethylated and individuals with Weaver were hypomethylated, compared to neurotypical controls and individuals with Sotos syndrome.

Figure S2. Pathogenic variants in *ASXL1* are associated with a distinct DNAm signature. (A) Principal component analysis (PCA) and (B) heatmap showing clustering of BOS individuals with truncating *ASXL1* variants (n=14; yellow), individuals with missense *ASXL1* VUS (n=3; red), the individual with the truncating *ASXL2* variant (n=1; purple), individuals with truncating *ASXL3* variants (n=3; blue) and control discovery subjects (n=26; grey) using DNAm values at the 763 CpG sites identified in the BOS DNAm signature. The heatmap color gradient indicates the normalized DNAm value ranging from -2.0 (blue) to 2.0 (yellow). Euclidean distance metric is used in the heatmap clustering dendrograms.
